# Prevalence-dependent use of serological tests for diagnosing glanders in horses

**DOI:** 10.1186/1746-6148-5-32

**Published:** 2009-09-01

**Authors:** Lisa D Sprague, Reena Zachariah, Heinrich Neubauer, Renate Wernery, Maria Joseph, Holger C Scholz, Ulrich Wernery

**Affiliations:** 1 OIE Reference Laboratory for Glanders, Friedrich-Loeffler-Institute, Naumburger Str. 96a, D-07743 Jena, Germany; 2 Central Veterinary Research Laboratory, P.O. Box 597, Dubai, United Arab Emirates; 3 Institute of Microbiology of the German Armed Forces, Neuherbergstr. 11, D-80937 München, Germany

## Abstract

**Background:**

The internationally mandatory complement fixation test (CFT) for testing of equine sera for the absence of glanders has repeatedly led to discrepant results. Not only do "false positive" sera pose a problem for the diagnostician and the animal health authorities but they can also result in significant financial losses for the animal owners.

Due to the very low prevalence of glanders in the horse population it is of major importance to use tests with a high specificity to overcome unreliable predictive values. We have compared formalin-fixed *B. mallei *whole cell antigen and a well characterised mouse monoclonal antibody with regard to their specificity and sensitivity for glanders serodiagnosis using CFT, an indirect (i) and a competitive (c) ELISA platform.

**Results:**

Our results show that the CFT is still a very reliable technique in horse populations with very low glanders prevalence. The cELISA has a high sensitivity and specificity comparable to that of the CFT. The cELISA offers the possibility for automatisation, can be applied to non-complement fixing sera and used for various host species.

**Conclusion:**

The CFT is still the method of choice for testing horses for the absence of glanders.

## Background

Although glanders has allegedly been eradicated from most countries of the western hemisphere, it is endemic among domestic animals in Africa, Asia, the Middle East and Central and South America [1,2]. Occasional reports on re-introduction of glanders into disease-free regions, e.g. Germany, exist [3]. The etiologic agent of glanders is *Burkholderia mallei*, a Gram-negative, nonmotile, facultative intracellular bacterium. Horses are highly susceptible to infection and considered to be the natural reservoir, but also mules and donkeys succumb to infection [1]. Glanders affects the upper respiratory tract and the lungs which develop granulomas that evolve into ulcers. Further symptoms are purulent nasal discharge, pneumonia and poor general condition. The cutaneous form (farcy) appears on the surface of limbs and body. Subclinically and latently infected carriers spread the bacterium under crowding conditions and stress, via infected food and drinking water or commonly used harnesses.

Within eradication programs the complement fixation test (CFT) has been the favoured serological diagnostic tool thanks to its ability to detect clinically in-apparent carriers. Although negative reactions are occasionally observed in sera of old, pregnant or worn out animals, and false positive reactions occur in approx. 1% of the tested horse sera (with hindsight attributable to the use of whole cell antigen) the CFT has an excellent sensitivity of at least 97% when compared to the gold standard pathology [4]. But in times of low disease prevalence more emphasis has been placed on developing tests with higher specificity in order to avoid false positives responsible for unnecessary restrictions in international animal transport.

When the prevalence is below 0.07% only tests with specificities of 99.995% provide reasonably accurate identification of the disease [5]. Hence, several techniques, such as counter-immunoelectrophoresis (CIET), western blot, Rose Bengal test (RBT) and competitive ELISA (cELISA), which were believed to meet this requirement, were thoroughly investigated. However, none of these tests fulfilled this criterion [6-9]. These tests are either based on crude bacterial preparations or uncharacterised monoclonal antibodies. A recent re-evaluation study on the performance of the four serological techniques CFT, IHAT (indirect hemagglutination test), RBT and modified CIET showed that no significant improvement of sensitivity is achieved when using crude whole cell antigens on prevailing materials or currently circulating strains [10]. Consequently, in order to improve test quality new analytical test substances need to be evaluated with particular emphasis on their immunogenetic characterisation and their degree of purity.

This paper describes the use of a formalin-fixed *B. mallei *whole cell antigen, and the well characterised *B. mallei *specific monoclonal antibody 3D11 in an indirect and a competitive ELISA for the serodiagnosis of glanders in comparison to the CFT.

## Methods

Eight hundred and six sera from imported horses and horses due for export were included in this study and divided into five groups (Table 1). Group I: 732 glanders-free horses originating from various countries; group II: ten suspect cases; group III: five horses in contact with horses with acute glanders; group IV: 34 horses due for import or export, of which two developed glanders; group V: 25 malleinised horses previously in contact with a horse in group II. Mallein was purchased from the Central Veterinary Control and Research Institute (Etlik, Ankara, Turkey) and 0.2 ml were injected intracutaneously (i.c.) into the left side of the neck. No skin reaction was observed after malleinisation. The sera were stored at the Central Veterinary Research Laboratory (CVRL), Dubai, in 2 ml Eppendorf tubes and maintained for several years at -20°C prior to testing.

**Table 1:** Comparison of the results from 806 sera investigated by means of complement fixation test, indirect and competitive ELISA

			**CFT**				**iELISA**			**cELISA**		
**Group**	**Origin**	#	**ccPro**		**Dubai7**		**-OD** **<0.125**	**OD** **0.125-0.185**	**+OD** **>0.185**	**-OD** **>0.8**	**OD** **0.775-0.800**	**+OD** **≤ 0.775**
			**+**	**-**	**+**	**-**						
I	Bavaria	100	0	100	0	100	65	18	17	86	3	11
	Syria	164	0	164	0	164	124	18	22	159	2	3
	Lebanon	15	0	15	0	15	13	1	1	15	0	0
	Qatar	56	0	56	0	56	44	7	5	54	0	2
	Saudi Arabia	52	0	52	0	52	34	10	8	50	0	2
	Kuwait	52	0	52	0	52	40	8	4	51	1	0
	Kish	12	0	12	0	12	8	0	4	12	0	0
	Mauritius	32	0	32	0	32	18	8	6	32	0	0
	Bahrain	49	0	49	0	49	22	14	13	49	0	0
	Dubai	82	0	82	0	82	74	6	2	81	0	1
	Jordan	51	0	51	0	51	34	6	11	11	0	0
	Egypt	57	0	57	0	57	42	13	2	56	0	1
	Oman	10	0	10	0	10	7	3	0	10	0	0
II	Syria	10	0*	9	3	7	7	0	3	7	0	3
III	Al Ain, Dubai, (contact cases)	5	4	1	5	0	0	1	4	0	0	5
IV	Miscellaneous	34	dubious reactions				17	3	14	32	0	2
V	Malleinised horses	25	for details see Table 2									

TOTAL		806										

*1 dubious reaction

### Complement fixation test and antigen preparation

All sera were tested by the recommended CFT according to the OIE Manual of Diagnostic Tests and Vaccines for Terrestrial Animals [9] using the commercially available CFT (ccPro, Neustadt/W., Germany) and an in-house CFT employing the antigen from a virulent *B. mallei *strain (Dubai7) isolated during a glanders outbreak in Dubai in 2004 [11]. The ccPro antigen is produced according to the standards set by the OIE Manual of Diagnostic Tests and Vaccines for Terrestrial Animals [9]. Samples were considered negative when 100% haemolysis occurred at a 1:5 dilution, 25-75% haemolysis were considered as dubious and no haemolysis was considered as positive [9].

Dubai7 was grown on blood agar for 72 hours. The colonies were suspended in 0.9% NaCl, treated with 37% formalin overnight, washed with PBS, pelleted and diluted in PBS (OD 0.2). The preserved stock antigen was maintained at -20°C at the CVRL. Positive control serum was obtained from ccPro.

### Selection of cut-off values for the indirect and the competitive ELISA

For the indirect ELISA, a cut-off was selected which was based on the OD value observed for the weakest positive horse. No bacterium was isolated but this animal was positive in a *B. mallei *specific PCR from formalinised tissue [11]. The CFT titre of this horse was 1:20 ++ with the commercially available ccPro antigen and 1:40++ with the Dubai7 antigen. The OD value of this sample was 0.180 and was used in this ELISA as the weak positive control. This finding resulted in the conclusion that sera showing an OD value between 0.125 - 0.185 (round off values 0.05) could be considered as dubious reactors thus requiring confirmatory analysis. The strongly reacting positive serum came from a confirmed glanders case from which the bacterium was isolated. The OD value of this sample was 0.785. The serum showed a CFT titre at 1:40++++ with the ccPro antigen and 1:80+++ with the Dubai7 antigen. Necropsy revealed positive glanderous lesions. The PCR results from the tissues were also positive for *B. mallei*. The negative sample came from an equine foal, which tested negative in all three tests with the lowest OD value of 0.050.

The same control sera were used for the competitive ELISA. The strong positive control showed an OD value of 0.097 and the weak positive control showed an OD value of 0.750, the negative control showed an OD value of 1.135. The cut-off was set at 0.775 (0.750+0.025 = 0.775) to include putative reactors falling within this range. OD values in the range 0.775 - 0.800 were considered as dubious reactors.

### Serum analysis by means of an indirect ELISA

Subsequently, the sera were tested in an indirect ELISA (iELISA) using the Dubai7 formalinised antigen preparation at a dilution of 1:2,000. Equine sera and the anti-horse conjugate (Sigma, Taufkirchen, Germany) were diluted to 1:1,000 and 1:10,000 respectively. The following buffers were used: wash buffer 0.05% PBST, and dilution buffer 0.05% PBST + 5% milk powder and 1% bovine serum albumin. The anti-horse IgG HRP conjugate and substrate TMB (3,3',5,5'-tetramethylbenzidine with sodium perborate substrate buffer) were obtained from Sigma (Taufkirchen, Germany). All reactions were stopped with 1 M sulphuric acid. All serum evaluations were measured at 450 nm and readings were taken on the SUNRISE™ Tecan machine (Crailsheim, Germany).

### Serum analysis by means of an in house competitive ELISA

Sera were then analysed by means of an in-house cELISA using the Dubai7 formalinised antigen preparation at a dilution of 1:500. Equine sera and the anti-horse conjugate (Sigma, Taufkirchen, Germany) were diluted to 1:1,000 and 1:10,000, respectively. The mouse monoclonal antibody 3D11 (Biotrend, Köln, Germany) was diluted 1:500. This monoclonal antibody derives from the hybridisation of SP2/0 myeloma cells with spleen cells of Balb/c mice immunised with a cell extract of *B. mallei*. This well characterised antibody targets a *B. mallei *specific epitope of the OPS [12,1]. The following buffers were used: washing buffer 0.01 M PBS and dilution buffer 0.01 M PBS with 0.1% Tween20™ and 0.3% bovine serum albumin. The anti-mouse HRP conjugate was used at a dilution of 1:10,000 and visualised with the substrate OPD (o- phenylenediamine) (both Sigma, Taufkirchen, Germany). The reactions were stopped with 1 M sulphuric acid. All readings were taken at 492 nm using the SUNRISE™ Tecan machine.

## Results

In Group I, 100% specificity was observed for all samples tested with the CFT and both antigens (ccPro and Dubai7) (Table 1). In group II, no positive results were registered with the ccPro antigen, 10% of the samples gave dubious results and the rest (90%) was negative. The Dubai7 antigen registered three positive but no dubious results. In group III, 80% positive and 20% negative results were observed for the samples tested with the ccPro antigen and 100% were positive with the Dubai7 antigen. In group IV, all sera of the horses showed dubious reactions in the CFT ranging from 1:2 - 1:10. The results obtained were independent of the antigen used (ccPro and Dubai7 antigen). In group V (Table 2), both CFTs tested horses identically positive or negative. Prior to malleinisation all 25 horses were negative (ccPro and Dubai7 antigen). At day 17, 7 horses (3, 10, 11, 16, 17, 20, 25) were negative. At day 42, 13 horses were still positive. On day 134, 1 horse (11) was positive in the CFT and 2 horses (19, 22) had dubious results.

**Table 2:** Detection of antibodies against mallein by means of serological assays over a period of 19 weeks

	**CFT**	**iELISA**	**cELISA**	**CFT**	**iELISA**	**cELISA**	**CFT**	**iELISA**	**cELISA**	**CFT**	**iELISA**	**cELISA**
Group V	ccPro/ Dubai7	Dubai7	Dubai7/ 3D11	ccPro/ Dubai7	Dubai7	Dubai7/ 3D11	ccPro/ Dubai7	Dubai7	Dubai7/ 3D11	ccPro/ Dubai7	Dubai7	Dubai7/ 3D11
horse	Day 0			Day 17			Day 42			Day 134		
1	-	-	-	+	+	-	+	+	-	-	-	-
2	-	-	-	+	-	-	-	+	-	-	-	-
3	-	-	-	-	-	-	+	-	-	-	-	-
4	-	-	-	+	+	+	+	-	-	-		-
5	-	-	-	+	-	-	-	-	-	-	-	-
6	-	-	-	+	-	-	+	-	-	-	-	-
7	-	-	-	+	-	+	+	-	-	-	-	-
8	-	-	-	+	-	+	+	-	-	-	-	-
9	-	-	-	+	+	-	+	-	-	-	-	-
10	-	-	-	-	-	-	-	-	-	-	-	-
11	-	-	-	-	-	-	+	-	-	+	-	-
12	-	-	-	+	+	+	+	-	-	-	-	-
13	-	-	-	+	-	-	+	-	-	-	-	-
14	-	-	-	+	-	-	-	-	-	-	-	-
15	-	-	-	+	-	-	+	-	-	-	-	-
16	-	-	-	-	-	-	-	-	-	-	-	-
17	-	-	-	-	-	-	-	-	-	-	-	-
18	-	-	-	+	-	+	-	-	-	-	-	-
19	-	-	-	+	-	-	-	-	-	1:2	-	-
20	-	-	-	-	+	-	-	-	-	-	-	-
21	-	-	-	+	+	-	+	-	-	-	-	-
22	-	-	-	+	+	+	-	+	+	1:2	+	+
23	-	-	-	+	-	-	+	-	-	-	-	-
24	-	-	-	+	+	-	-	-	-	-	-	-
25	-	-	-	-	-	-	-	-	-	-	-	-

+: positive reaction;

-: negative reaction;

In the iELISA using the Dubai7 antigen the following results were observed: In group I, 112 (15.3%) samples showed doubtful results, 526 (71.8%) were negative and 94 (12.9%) gave false positive results (Table 1). After excluding the 100 Bavarian samples because of lipid contamination, 94 (14.9%) dubious and 78 (12.4%) false positive results were recorded. In group II, the iELISA revealed 3 (30%) positive, and 7 (70%) negative results. In group III, 4 (80%) samples were positive, and 1 (20%) was dubious. In group IV, 14 (41.2%) positive, 3 (8.8%) dubious and 17 (50%) negative samples were found. In group V, all 25 horses were negative and below the cut-off value (0.185) at day 0 prior to malleinisation. At day 17, horses 1, 4, 9, 12, 20, 21, 22 and 24, and at day 42, horses 1, 2 and 22 were positive. On day 134, horse 22 was still positive (Table 2). The cut-off for the iELISA was based on the OD value observed for the weakest positive Syrian horse in group II (0.180). The negative sample derived from an equine foal which tested negative in the CFT, iELISA and cELISA. The OD value was 0.050.

Analyses of the samples using the cELISA with the Dubai7 antigen and the mouse monoclonal antibody 3D11 gave following results: In Group I, 6 (0.8%) samples showed doubtful results, 706 (96.5%) were negative and 20 (2.7%) gave false positive results (Table 1). Upon exclusion of the 100 Bavarian samples, 3 (0.47%) dubious and 9 (1.42%) false positive results were recorded. In group II the cELISA revealed 3 (30%) positive, 7 (70%) negative and no dubious results. In group III, 5 (100%) positive results were recorded. In group IV, 2 (5.9%) positive, 32 (94.1%) negative but no dubious results were found. In group V, 25 sera were negative at day 0 prior to malleinisation. At day 17, 6 horses (3, 6, 7, 12, 18, 22) were positive. At day 42 and 134, 1 horse (22) was still positive (Table 2). The same control sera used in the iELISA was also applied to the cELISA. The strong positive control showed an OD value of 0.097 and the weakest positive control showed an OD value of 0.750. The negative control showed an OD value of 1.135. The graphically deduced cut-off was set at 0.775 (0.750 + 0.025 = 0.775) to include putative reactors falling within this range. OD values in the range 0.775 - 0.800 were considered as dubious reactors.

## Discussion

In recent years laboratories with high numbers of equine sera for import/export testing have encountered discrepant glanders results when using the internationally mandatory CFT. Wernery et al. [13], for instance, found 0.12% positive reactors in 22,212 sera from healthy horses tested over a ten year period in the United Arab Emirates. These reactors pose a logistical problem not only to the investigator but also to the animal health authorities and to the horse owner. Due to the assumed very low prevalence in the horse population it is of major importance to use tests with a high specificity to overcome unreliable predictive values. We therefore investigated the influence of formalin-fixed *B. mallei *whole cell antigen (ccPro; Dubai7) and a well characterized mouse monoclonal antibody (3D11) with regard to their specificity and sensitivity for glanders serodiagnosis using an indirect and a competitive ELISA platform and compared it to the CFT. The Dubai7 antigen used in both ELISA was produced by formalin incubation.

The outcome of the results for the samples from "glanders free" horses (group I) in the indirect ELISA was unexpected with regard to specificity (Tables 1 and 3), in particular in the Bavarian horse samples obtained from a "disease free zone". These false positive reactions could be attributed to the fact that the latter sera were of poor quality due to lipemic plugs. These products possibly affected the test by non-specific blocking of antibody binding sites. These findings also applied to the cELISA, whereas both CFT were not affected by the quality of the sera. Therefore, we decided to exclude the Bavarian samples for calculating specificity (Table 3).

**Table 3:** Group wise comparison of sensitivity (SN) and specificity (SP) between the serological assays in groups I-IV (figures in brackets include the Bavarian samples)

		**CFT**				**iELISA**		**cELISA**	
		ccPro		Dubai7		Dubai7		Dubai7/3D11	
group	#	SN (%)	SP (%)	SN (%)	SP (%)	SN (%)	SP (%)	SN (%)	SP (%)
I	632 (732)	-	100	-	100	-	85,5 (84,6)	-	98,5(97,2)
II	10	50	100	100	100	100	100	100	100
III	5	80	-	100	-	80	-	100	-
IV	34	-	-	-	-	100	58,6	100	100

In group II ("suspected glanders") one serum tested 'dubious' in the ccPro CFT and was initially considered to have reacted 'non specific', however, it tested positive with the Dubai7 antigen. This serum and two further sera reacted positive in both ELISA. These serologically positive horses were confirmed to be glanderous by PCR, whereas the remaining seven horses were serologically and PCR-negative. Interestingly, this time the iELISA was as sensitive as the cELISA and more sensitive than the CFT. The most likely explanation for the failure of the CFT is that during the manufacturing process the number of reactive epitopes is reduced by destruction or blockage. Consequently, sera with low specific antibody titres are not identified. We do not believe that *B. mallei *strains belonging to different 'serogroups' or strains displaying a considerable number of strain specific epitopes are in circulation [1]. Genetic mutations during subcultivation in artificial media result in the loss of LPS or CPS expression [1].

In group III ("contact cases") we observed that one serum of a horse going through the active phase of disease was not recognised as positive in the commercial (ccPro) CFT but showed high titres with the Dubai7 antigen (1:160+ to 1:320+). This false negative result might be caused by technical problems during the manufacturing process e.g. low quality of the antigen.

The samples of group IV ("miscellaneous") represent the classical serum panel frequently encountered when testing for import/export procedures (Table 1). These samples were collected over a 10 year period. All the sera from this group showed titres ranging from 1: 2 to 1:10 in both CFT; hence they were classified as dubious. Although both ELISA use the Dubai7 antigen preparation, only the cELISA showed 100% specificity (Table 3). The high numbers of dubious and false negative reactions in the iELISA are most likely due to cross reactions with other bacterial species with similar cell wall antigens [1,14]. This cross reaction has been overcome by the cELISA due to the use of a monoclonal capture antibody (3D11). Only two out of 34 sera gave positive results. These sera came from an import horse originating from Syria and from a horse from Al Ain, UAE. Both were shown to be infected with *B. mallei *by PCR or culture, respectively. Thus, the cELISA clearly increases the sensitivity and specificity of disease diagnosis in dubious CFT cases.

In group V ("malleinised horses") all the applied tests were influenced by antibodies against mallein for up to 19 weeks after malleinisation. These findings are in accordance with Verma et al. [15]. Although horse 22 showed positive reactions in all serological assays up to week 19 we do not believe this animal to be affected by glanders since this animal was clinically inconspicuous, showed no sign of disease and did not react to the malleinisation. Therefore, another explanation for the reaction could be a so called 'sticky' serum. These sera are prone to unspecific reactions and have been observed by us and other working groups [7,13,16].

We do not have an explanation for the 100% specificity of the CFT technique in this study. It has always been reported to be around 99%, thus up to seven false positive results should have been found in the sera from the horses in group I. Due to its high specificity the CFT is still a very reliable technique especially in horse populations with very low glanders prevalence [5]. The cELISA also has a very promising sensitivity and specificity comparable to that of the CFT. The major advantages of the cELISA are its potential for automatisation and its applicability in cases of non-complement fixing sera. Moreover, like the CFT it can be used for various host species. We were able to test the cELISA on human serum from a laboratory infected person with good results (pre-infection serum: 1.009; post-infection serum: 0.443) [17].

## Conclusion

Although the CFT is an old and labour intensive technique, it still meets the requirements for diagnosing glanders in a low prevalence population. However, in order to avoid costly false positive results we recommend the combined use of CFT and cELISA whenever possible.

## Abbreviations

CFT: complement fixation test; CIET: counter-immunoelectrophoresis; CPS: capsular polysaccharide; CVRL: Central Veterinary Research Laboratory; ELISA: enzyme-linked immunosorbent assay; cELISA: competitive ELISA; iELISA: indirect ELISA; HRP: horseradish peroxidise; IHAT: indirect hemagglutination test; LPS: lipopolysaccharide; OD: optical density; OIE: Office International des Epizooties (World organisation for animal health); OPD: o-phenylenediamine; PCR: polymerase chain reaction; RBT: Rose Bengal Test.

## Authors' contributions

LDS: evaluated and interpreted the data and wrote the manuscript; HN: designed the study and drafted the manuscript; RZ and MJ: developed and established the cELISA and carried out all serological assays; HCS: purified and characterised the Dubai7 antigen; RW and UW: collected the serum samples, malleinised and health monitored the horses. All authors revised the manuscript critically and approved the final version.
